# POLICY SERIES: INTERDISCIPLINARY PUBLIC POLICY DISCUSSION SESSION

**DOI:** 10.1093/geroni/igz038.1453

**Published:** 2019-11-08

**Authors:** Linda K Harootyan, Brian W Lindberg

**Affiliations:** 1 Wilmington, North Carolina, United States; 2 The Gerontological Society of America, Washington, District of Columbia, United States

## Abstract

This session, organized by the GSA Public Policy Committee, will provide both GSA section leadership and attendees an opportunity to have an open dialogue on important public policy issues of significance to the aging population. Section leaders will discuss key policy issues of particular relevance to their section’s mission and purpose. They also will comment on improving physical and mental health to illustrate how their different disciplines and perspectives inform and apply to public policy on that issue. This will be an interactive session with plenty of opportunity for audience feedback and input.

